# Applying the ^15^N labelling technique to material derived from a landfill simulation experiment to understand nitrogen cycle processes under aerobic and anaerobic conditions

**DOI:** 10.1007/s10532-022-10000-7

**Published:** 2022-10-11

**Authors:** Nora Fricko, Wolfgang Wanek, Johann Fellner

**Affiliations:** 1 Institute for Water Quality and Resource Management, TU Wien, Karlsplatz 13/226, 1040 Vienna, Austria; 2 Center of Microbiology and Environmental Systems Science, Department of Microbiology and Ecosystem Science, University of Vienna, Djerassiplatz 1, 1030 Vienna, Austria

**Keywords:** Landfill aeration, Landfill aftercare, ^15^N isotope pool dilution assay, Ammonium, Nitrate, ^15^N natural abundance

## Abstract

Reactive nitrogen (N) species, such as ammonium (NH_4_^+^), nitrate (NO_3_) and gaseous nitrous oxide (N_2_O), are released into the environment during the degradation of municipal solid waste (MSW), causing persistent environmental problems. Landfill remediation measures, such as in-situ aeration, may accelerate the degradation of organic compounds and reduce the discharge of ammonium via leachate. Nonetheless, the actual amount of N in the waste material remains relatively constant and a coherent explanation for the decline in leachate ammonium concentrations is still lacking. Hence, the present study aimed to elucidate the dynamics of N and its transformation processes during waste degradation. To this end, the gross rates of organic N mineralization and nitrification were measured using ^15^N pool dilution in waste material derived from a landfill simulation reactor (LSR) experiment. The results revealed a high potential for N mineralization and nitrification, the latter of which declined with the diminishing amount of extractable ammonium (after aeration). The analysis of the concentration and isotopic composition of N_2_O formed confirmed incomplete denitrification as the main source for N_2_O. Moreover, the natural abundance of ^15^N was investigated in various waste N pools to verify the conclusions drawn from the ^15^N tracing experiment. δ^15^N values of total waste N increased during aeration, indicating that nitrification is the major driver for N losses from aerated waste. The application of stable isotopes thereby allowed unprecedented insights into the complex N dynamics in decomposing landfill waste, of their response to aeration and their effect on hydrological versus gaseous loss pathways.

**Supplementary Information:**

The online version contains supplementary material available at 10.1007/s10532-022-10000-7.

## Introduction

Steffen et al. (2015) defined planetary boundaries as the environmental limits within which humanity can safely operate. They identified the global nitrogen cycle as one of the planetary boundaries, where anthropogenic changes will exceed these boundaries, which might push the Earth system into a new state. The main driver for this development relates to industrial nitrogen fixation for the production of nitrogenous fertilizers in agriculture. However, other processes also release reactive nitrogen into the environment, e.g. fossil fuel burning and biomass incineration. This also applies to biological degradation of municipal solid waste in landfills and dumpsites, resulting in the release of reactive nitrogen species like ammonium (NH_4_^+^) via hydrological pathways into landfill leachate (Berge et al. 2005), and ammonia (NH_3_) and nitrous oxide (N_2_O) into the atmosphere via gaseous pathways.

Whereas Europe enforced strict regulations (Council of the European Union 1999) that banned the landfilling of untreated municipal solid waste (MSW), the majority of the world’s countries continue this practice (Kaza et al. 2018). As the global amount of waste is constantly increasing, fueled by the growth of the human population and increased waste production per capita, and since nitrogen emissions emanating from landfills continue for decades after closure, the problem has not yet reached its peak. Following the closure of MSW landfills, remediation measures are often required to keep the discharge of hazardous substances via the gas and the liquid phase at an acceptable level. One of the frequently applied methods is in-situ aeration (Heyer et al. 1999), which is not only known for accelerating (aerobic) biomass decomposition and subsequently reducing methane emissions, but also causes a distinctive decline in ammonium release via leachate. Ammonium is not only known for being a persistent pollutant to surface and groundwaters, but also for inhibiting MSW degradation. Chamem et al. (2020) observed and described ammonium inhibition of waste degradation on a full-scale landfill level. The field application of in-situ aeration is, in many cases, combined with lab-scale studies (Hrad et al. 2013; Ritzkowski et al. 2016) utilizing landfill simulation reactors (LSR) to quantify the emission reduction achievable. Results from such studies (Brandstätter et al. 2015; Fricko et al. 2021) indicate that most of the nitrogen remains in the solid phase. This somehow contrasts with the observed decline in ammonium concentrations in the leachate. Additionally, the emission reduction seems not to be the result of enhanced incorporation into microbial biomass. To ensure that the ammonium emissions do not recur when the aeration is terminated, it is necessary to quantify the nitrogen transformation processes within the waste material in more detail and to trace nitrogen processes during waste degradation, which represents the objective of the present paper.

For the given objective, nine landfill simulation reactors were constructed and operated in triplicates, which were characterized by different (or non-existent) aeration modes. Samples retrieved from the reactors were incubated using ^15^N stable isotope labeling (isotope pool dilution approaches) to quantify gross rates of ammonium and nitrate production and consumption. Gas samples from the headspaces were analyzed for ^15^N_2_O. Subsequently, gross reaction rates were calculated. For verification purposes, natural ^15^N abundance data from unlabeled MSW materials and inorganic nitrogen pools in the experiment as well as bulk concentration in the solid phase (TN) and extractable ammonium and nitrate were investigated.

## Materials and methods

Thoroughly mixed waste material was taken from a MSW landfill dating back to the 1970s during the drilling of new gas wells. The landfill itself is located north-east of the city of Vienna, Austria, and is still under operation. Nonetheless, at the site also exist some older compartments without base layer where groundwater needs to be maintained below the usual level to avoid pollution by leachate. Due to the long period of closure of those compartments, the waste material is thought to contain fewer plastics and a higher share of biodegradable waste than nowadays. This material was kept in nine landfill simulation reactors (LSR) over a period of 25 months. All reactors experienced an initial anaerobic phase lasting 2 months to ensure equal starting conditions. Three reactors were then kept anoxic throughout the experiment. Six of the reactors were then aerated; for three of these reactors, aeration was terminated after 12 months, switching them back to an anaerobic regime. Hence, three distinct groups of reactors can be specified depending on the availability of oxygen over the experiment’s duration: a completely aerobic set, an aerobic set returning to anaerobic degradation and a completely anaerobic set. These groups are hereinafter referred to as “aerobic”, “aerobic–anaerobic” and “anaerobic”.

### LSR experiment

As the detailed design and a schematic diagram of the LSRs has already been published (Fricko et al. 2021), only the most important facts will be presented in this study. The reactors contained a total volume of 60 L; however, only about 40 L were initially filled with MSW. Leachate was collected at the bottom of the reactors and was recirculated (combined with exhaust gas measurements) every day at the beginning of the experiment (until day 275). In due course, the frequency of exhaust gas measurements and the leachate recirculation was reduced to three times a week. Generally, sampling of leachate was performed every 4 weeks. This interval decreased to 2 weeks after major changes in operation (such as starting or termination of aeration). The aeration gas (21% O_2_ in Ar 5.0, Messer, Austria) was introduced centrally into the waste material through an injection lance. The influx of aeration gas into each reactor was continuously measured and recorded. At first, the influx was kept constant at 30 mL min^−1^, but after 150 days of aeration (200 days of total operation) the influx was reduced to 15 mL min^−1^ due to strongly decreasing CO_2_ concentrations in the exhaust gas. At the times of leachate sampling, exhaust gas was sampled as well (into 2 × 20 mL GC-vials) and subsequently analyzed for N_2_O. The temperature of the reactors was measured with a PT-100 A sensor placed within the waste material. The corresponding signal was recorded and used for regulating a resistance heating, resulting in a mean temperature of 34.5 ± 1.2 °C.

Solid samples were retrieved from all reactors at the start and end of the experiment. For the aerobic and the aerobic–anaerobic group, additional sampling campaigns were performed at three distinct time points: at the start of aeration (approximately 2 months after experimental start, day 54 resp. 57), after 2 months of aerated operation (day 110 resp. 113) and after a longer period of stable aerobic operation (day 355 resp. 358). This sampling event also marked the return to anaerobic operation for the aerobic–anaerobic group. Hence, a total of 36 solid samples resulted. All solid samples were sieved to 4 mm and subsequently both fractions (≤ 4 mm and 4 mm) were analyzed for their elemental composition (carbon and nitrogen). Only samples from the smaller fraction (≤ 4 mm) were further utilized to perform the ^15^N pool dilution assays for mineralization and nitrification.

### ^15^N pool dilution assay

The method of isotope pool dilution assays refers to Kirkham and Bartholomew (1954) and makes use of the fact that: first the target pool is amended with an isotopically labeled tracer; second, the influx (production processes) into the labeled pool dilutes the isotopic tracer in it; and third, the efflux (consumptive processes) consumes the compound in the target pool at the isotopic enrichment as it prevails at any time point in the pool itself. Hence, if the size of the pool and its isotopic composition are determined at two distinct time points, a gross reaction rate for the production and consumption of this target compound can be estimated. As the production rate is determined from the product pool, it is considered to be unstimulated, whereas the addition of tracer (substrate) might increase the consumption rates (Booth et al. 2005). There are several assumptions underlying this approach: First, due to the high amendment of the heavier isotopes, any effects of isotopic fractionation can be neglected. Second, the isotopically labeled substance is assumed to be evenly distributed and the tracer is therefore understood to be in equilibrium with the native, unlabeled pool. Third, remineralization (e.g. by tracer uptake into microbes, death and microbial biomass decomposition) of the labeled substance is excluded. The last assumption complied by the relatively short incubation periods (Braun et al. 2018), but nonetheless all assumptions can possibly be violated under specific circumstances.

In the present study, isotope pool dilution assays were performed for mineralization of organic N and for nitrification. Thus, the labeled substances applied were ^15^NH_4_Cl for mineralization and K^15^NO_3_ for nitrification. Some of the steps required for sample preparation were independent of the analysis (either mineralization or nitrification), therefore they first receive mention below. Additionally, the procedure for sample preparation is summarized in Fig. S1. Immediately after sampling waste material from the LSRs and sieving, approximately 30 g of the fresh sieved sample were extracted with 2 M KCl for 1 h and subsequently analyzed for NH_4_-N and NO_3_-N (DIN EN ISO 13395 1996). Based on these measurements, the necessary volume and isotopic enrichment of tracer solution was calculated and accordingly prepared. The samples themselves were stored at 6 °C overnight. On the following day three batches for each sample derived from the reactors were prepared separately: one with tracer addition, which was terminated after 4 h, another one with tracer addition, which was terminated after 24 h, and a third one as an unlabeled reference without any tracer addition, which was also incubated for 24 h. Each of these batches required about 4 g of waste material from the fraction ≤ 4 mm. The isotope pool dilution assays were adjusted depending on group and time point of sampling due to large changes in ammonium and nitrate concentrations necessitating adjustments in the volume and isotopic enrichment of the tracers (indicated in the supplementary information, Tables S1 and S2 by “pool” and “spike”). Adding approximately 10% of the initial, native pool size as ^15^N tracer at 98 at%^15^N enrichment to the waste samples (referred to as “pool”) was the target if the pool size of ammonium or nitrate exceeded a sensible size. If the existing pool size was too low for later isotope analysis, a mixture of approximately 10% tracer and 90% unlabeled equivalent (“spike”) was dissolved in deionized water and applied to the sample to increase the target pool size to the very lowest measurement range of isotope pool dilution assays. For nitrification, this approach had to be conducted for all sampling campaigns due to continuously low nitrate levels. To prove that the amount of nitrogen applied in this manner does not bias isotope pool dilution, four different concentrations of spikes (see supplementary information, Table S3) were tested during the sampling campaign at day 355 (resp. 358). Continuing with the incubation procedure, 1 mL of either tracer solution or spike (see Tables S1, S2) were added to the respective material. After the corresponding time periods, the incubations (room temperature) were terminated with an addition of 30 mL 2 M KCl to the sample. The vials were placed on an orbital shaker (200 rpm, 30 min) and subsequently filtered through ash-free cellulose filters. The extracts were stored at − 20 °C until further analysis. The subsequent procedure (microdiffusion) for ammonium in terms of measuring mineralization of organic N and for nitrate for nitrification differed, but the extracts were never consumed completely. Hence, the remaining extracts were subsequently stored at − 20 °C to enable technical replication as well as cross-checking results obtained from microdiffusion, with measurements of NH_4_-N and NO_3_-N in the extracts according to (DIN EN ISO 13395 1996). Moreover, several blanks (10 mL 2 M KCl, with no waste material or tracer addition) were run through the whole procedure to account for the nitrogen impurities of the chemicals used.

#### Mineralization

Following incubation and extraction, ^15^N pool dilution assays for mineralization (Division of Terrestrial Ecosystem Research, 2017a) were conducted as follows: the extracts were diluted with 2 M KCl to the optimal range of 10–25 (up to 70) µg NH_4_-N 10 mL^−1^ and 10 mL of the diluted extracts were transferred into 20 mL polypropylene vials filled with 100 mg MgO (triggers pH adjustment to the alkaline range). An acid trap (4 µL 2.5 M KHSO_4_ on disks of ash-free cellulose filter paper wrapped in a Teflon tape) was placed inside each vial. The vials were shaken for 2 days (200 rpm) to enable the diffusion of ammonia (high pH shifts equilibrium between ammonium and ammonia towards ammonia) out of the solution into the acid trap, where ammonia is completely trapped as ammonium under acidic conditions. Subsequently, the acid traps were removed and dried in a desiccator over concentrated H_2_SO_4_ for 24 h. The acid traps were prepared for EA-IRMS (elemental analyzer Flash-EA coupled via a ConFlo III interface with a Delta V Advantage isotope-ratio mass spectrometer, Thermo Scientific) by removing the Teflon tape and wrapping the filter disks into tin capsules.

#### Nitrification

In contrast to the procedure for mineralization, ^15^N pool dilution assays for nitrification (Division of Terrestrial Ecosystem Research 2017b) require an additional step: the complete removal of NH_4_-N from the extract since NO_3_-N is then reduced with Devarda’s alloy under alkaline conditions to ammonium and subsequently captured and measured as NH_4_-N. In anaerobic waste material, NH_4_-N by far exceeds NO_3_-N, hence the complete removal of ammonium represented a major effort. The following steps were carried out for measuring gross nitrification: similar to the procedure for mineralization, the (diluted) extract (10 mL) was amended with 100 mg MgO in 20 mL polypropylene vials to increase the pH of the extract and to remove ammonium as ammonia. To ensure complete removal, up to 15 acid traps were added in this step and the vials closed. The vials were placed on an orbital shaker (200 rpm) for 2 days. Subsequently, the acid traps were discarded and 50 mg of Devarda’s alloy was added along with a new acid trap. Again, the vials were shaken (200 rpm) for 2 days. Finally, the acid trap was taken out and dried in a desiccator for 24 h. The acid traps were prepared for EA-IRMS in the same manner as for mineralization. Due to the low nitrate concentrations prevalent, all samples except for three were spiked with 1.3–1.8 mg K^15^NO_3_ and 13.2–13.9 mg KNO_3_ dissolved in 10 mL of deionized water to initiate the isotope pool dilution assays. The three exceptions are explained easily: Nitrate concentrations are expected to increase during the aeration of old MSW landfills. However, with a remarkably long delay (Brandstätter et al. 2015; Fricko et al. 2021). Hence, only for the completely aerobic reactors at the very end of the experiment did nitrate concentrations reach a value high enough for direct labeling of the pool instead of spiking. The dilution applied to the nitrification extracts varied from 25 at the beginning of the experiment to 5 at the end — depending, in particular on the concentration of ammonium which had to be removed in the first place. Details on the extracts and dilutions are displayed in the supplementary information Table S2.

#### Isotope pool dilution calculations

Although the sample preparation steps differed for mineralization and nitrification, the subsequent calculations to obtain gross reaction rates were equal (Division of Terrestrial Ecosystem Research 2017a, b). First, the measured atom percentage of ^15^N ($at{\%}^{15}{N}_{meas}$) was blank corrected (${at\%}^{15}{N}_{blk}$) using the total amount of nitrogen within the sample and the blank (undiluted ${N}_{meas}$ and ${N}_{blk}$ in µg) resulting in the true atom percentage of ^15^N in the sample ${at\%}^{15}{N}_{true}$:

1 $${at\%}^{15}{N}_{true}=\frac{{at\%}^{15}{N}_{meas}\cdot {N}_{meas}-{at\%}^{15}{N}_{blk}\cdot {N}_{blk}}{{N}_{meas}-{N}_{blk}}$$

The calculation for the corresponding gross reaction rates (“GP” refers to gross production, “GC” to gross consumption) was then continued with the blank corrected atom percentage (${at\%}^{15}{N}_{true}$) minus the atom percentage of the respective background samples ($at{\%}^{15}{N}_{bg}$, determined in the non-labeled incubation samples), yielding the atom percentage enrichment above the natural ^15^N abundance (APE, here: ${at\%}^{15}{N}_{t}$). The background samples themselves were not blank corrected as the differences in the amount of nitrogen between blank and background were too small.

2 $${at\%}^{15}{N}_{t}={at\%}^{15}{N}_{true}-{at\%}^{15}{N}_{bg}$$

3 $$GP=\frac{{c}_{t2}-{c}_{t1}}{{t}_{2}-{t}_{1}}\cdot \frac{\text{l}\text{n}\left(\frac{at{\%}^{15}{N}_{t1}}{{at\%}^{15}{N}_{t2}}\right)}{\text{l}\text{n}\left(\frac{{c}_{t2}}{{c}_{t1}}\right)}$$

4 $$GC=\frac{{c}_{t1}-{c}_{t2}}{{t}_{2}-{t}_{1}}\cdot \left[1+\frac{\text{l}\text{n}\left(\frac{{at\%}^{15}{N}_{t2}}{{at\%}^{15}{N}_{t1}}\right)}{\text{l}\text{n}\left(\frac{{c}_{t2}}{{c}_{t1}}\right)}\right]$$

The indices 1 and 2 correspond to the time at which incubation was terminated (4 h resp. 24 h). The concentrations ${c}_{t1}$ and ${c}_{t2}$ are given in mg N kg DM^−1^. Data on concentrations obtained by optical measurements (DIN EN ISO 13395 1996) of the extracts were considered here rather than the measurements from microdiffusion. If at%^15^N_t2_ exceeded at%^15^N_t1_, the samples were excluded from subsequent data calculations.

#### ^15^N_2_O

The gas formed in the headspace of the incubation vials of each sample during the isotope pool dilution assay was analyzed with respect to the concentration and isotopic composition of N_2_O, regardless of tracer addition or incubation time. Hence, all incubation vials were purged with Ar 5.0 or aeration gas (depending on operation mode) directly after tracer addition. Before terminating the incubation with 2 M KCl, the gas formed during incubation was extracted with a syringe and transferred to a GC-vial. The analysis itself was carried out at the Stable Isotope Facility (SIF), University of California (UC) Davis, California. Some of the gas samples had to be stored at room temperature before transportation and analysis (time between sampling and analysis could last up to 10 months). No technical replication of these gas samples was possible, but for cross-checking the results of the incubation samples a comparison between measurements of N_2_O concentration on reactor off-gas samples at SIF UC Davis and immediate measurements of N_2_O concentration on off-gas samples in-house (Research Unit for Water Quality Management) was conducted. This approach displayed acceptable deviations, and six obviously incorrect measurements were excluded from the interpretation. Due to leakage of the vials during storage, N_2_O tends to be underestimated after prolonged storage, e.g. as in the measurements performed by SIF UC Davis, which is confirmed (see supplementary information Fig. S2). The analysis was conducted according to the SOP of SIF UC Davis, version 2019, using a helium carrier stream and subsequent GC-IRMS analysis of the headspace samples.

### δ^15^N natural abundance and bulk concentrations

The preferred microbial transformation of isotopic lighter compounds causes an initial substrate (IS) to experience enrichment of heavier isotopes during the corresponding process. Hence, the remaining substrate (RS) is enriched, whereas the cumulative product (CP) gets depleted in heavier isotopes. Measuring the concentration and the ratios of natural isotopes (e.g. δ^15^*N *(‰) = $\left( {\frac{{^{{15}} N/^{{14}} N_{{Sample}} }}{{^{{15}} N/^{{14}} N_{{S\tan dard}} }} - 1} \right) \cdot 1000$, where the standard is atmospheric N_2_) in RS allows an estimation of the fraction f — the ratio of CP related to IS. To comply with the mass balance, f can’t exceed 1. Combined with the isotopic fractionation factors (Δ) of each individual process, a system of subsequent transformations (CP of the preceding process as input to the next one) can be described. Isotope fractionation modeling has recently been pioneered for nitrogen in soils by Xu et al. (2021).

Measuring natural isotopic fractionation (natural abundance) of carbon and nitrogen has become increasingly common for characterizing degradation processes in waste and compost. Wimmer et al. (2013) measured δ^13^C in leachates from differently operated MSW landfills and LSR experiments and obtained significant variations of δ^13^C in DIC with regard to the availability of oxygen. Lynch et al. (2006) documented changes in the δ^15^N signature of corn silage during composting.

In the present study, data regarding the isotopic composition of different N-species in non-labeled background samples was investigated: First, bulk nitrogen concentration (TN, mg kg DM^−1^) and the corresponding isotopic composition δ (‰) were measured directly in dried solid matter of each sieved reactor sample using EA-IRMS. Second, ammonium and nitrate concentrations and their corresponding δ values are of interest.

## Results and discussion

If not indicated otherwise, all results display the mean per operational group and errors are presented as standard error (1SE). The significance of deviations between the mean of an operational group and the corresponding sampling event were tested using a one-dimensional ANOVA and a subsequent Tukey-HSD test with a 95% confidence interval (supplementary information Tables S4, S5).

### ^15^N pool dilution assay

#### Testing different spike concentrations

Until day 355, six reactors (aerobic and aerobic–anaerobic) were aerated, resulting in a long period of stable aerobic degradation. Hence, the extracted amounts of NH_4_-N and NO_3_-N were around or below 1 mg L^−1^, too low for labelling 10% of the pool followed by isotope analysis by means of microdiffusion and EA-IRMS. Thus, four different spike concentrations (for details, see Table S3) were applied to the sieved waste material. In addition to the “standard” concentration of 15 mmol 10 mL^−1^ (thereof 10% ^15^N), two elevated concentrations (1.5 resp. two times the standard concentration) and 0.5 of the standard concentrations were examined. The test of different spike concentrations (see Fig. S3) revealed that consumption as well as production rates for ammonium were highest using the “standard” concentration. Consumption rates up to 40 mg N (kg DM day)^−1^ were observed, and production rates between 10 and 30 mg N (kg DM day)^−1^. For all other spikes, the rates were approximately half as high as for the “standard” concentration. For the lower spike, substrate limitation might have caused slower turnover. The microbiota might have become used to low available NH_4_-N due to continuous aeration. For nitrification, no specific pattern was observed, although a slight trend for higher rates [especially with regard to nitrate consumption, up to 60 mg N (kg DM day)^−1^] at higher spikes might be recognizable. This would indicate substrate limitation and a high adaption of the microbiota to nitrate assimilation. According to these results, the applied “standard” spiking concentration was considered reasonable in terms of manageable amounts as well as regarding the precision of the results obtained.

As mentioned previously, nitrate accumulates under aerobic conditions over the long term in the LSRs (and the aerated landfills). Hence, another opportunity to validate the spiking approach was the final sampling of the completely aerobic reactors at the end of the experiment at day 753. The NO_3_-N concentration had increased to 4 mg L^−1^, enabling pool labelling as well as spiking (concentration “standard”, approximately doubling the amount of nitrogen within the sample). The results are summarized in Fig. S4, displaying a mean consumption rate of 15 ± 5 mg N (kg DM day)^−1^ and a mean production rate of 5.8 ± 1.5 mg N (kg DM day)^−1^ calculated from the pool samples. However, the spiked samples showed negative results for nitrate consumption and only minimal production rates. As none of the spikes applied previously was added to an existing pool, the most probable explanation for the unexpected results from spiking are violations against the assumption of equilibrium between the native, unlabeled pool and the spike. Hence, the spiked samples from this specific sampling event were discarded and only the labeled ones considered hereinafter.

#### Gross mineralization

**Fig. 1:**
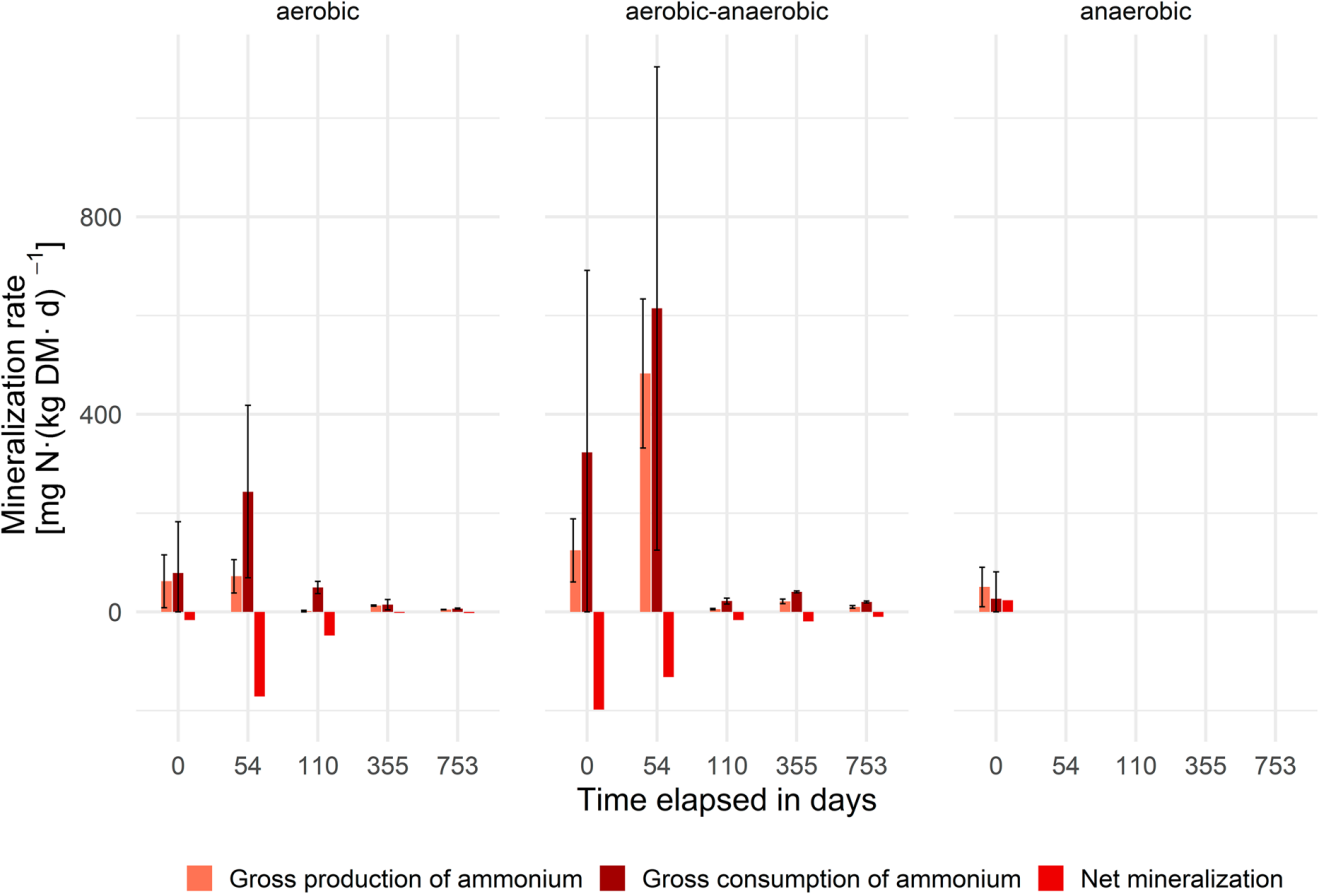
Mineralization rates per operation mode (n = 3) and experiment duration

Net mineralization is calculated as the difference of gross production of NH_4_-N (gross mineralization) minus gross consumption. All rates for the transformation of ammonium throughout the experiment are visualized in Fig. 1. Due to the restriction that in most final samples at day 753 at%^15^N_t2_ was greater than at%^15^N_t1_, which violates the assumptions of isotope pool dilution, only one measurement was considered valid for the completely anaerobic group at the end of the experiment. As no statistical evaluation is possible, the measurement is not displayed in Fig. 1. The most likely explanation for this singularly valid measurement is that the very high levels of ammonium concentrations over time in anaerobic reactors (~ 900 to 1000 mg N kg DM^−1^), while gross fluxes were small (~ 50 mg N (kg DM day)^−1^) compared to the large present reservoir, render the isotope pool dilution approach insufficiently sensitive to obtain quantifiable gross mineralization rates under such extreme conditions. The mean residence time here would approximate roughly 20 days, while Booth et al. (2005) indicated a mean residence time for ammonium of 1–3 days in terrestrial soils in a data synthesis, i.e. a much faster turnover of the soil ammonium pool and therefore faster isotope pool dilution rates.

As mentioned previously, the treatment was identical for the aerobic and the aerobic–anaerobic reactors until day 355, when aeration was terminated for the latter ones. Although the individual measurements between the groups differed strongly, for all of these reactors and all sampling campaigns, gross consumption of ammonium exceeded gross production (mineralization), thus resulting in a net removal of ammonium. The highest rates for both groups were observed at the beginning of the experiment (day 0) and before the start of aeration (day 54), which also represents the period of highest biodegradation (highest carbon turnover, Fricko et al. 2021). This observation reveals that there is a large potential for the consumption of ammonium during this period, which corresponds well with the decline in leachate NH_4_-N concentrations from 604 ± 22 to 0.40 ± 0.04 mg L^−1^ within 14 days after the start of aeration. However, it also demonstrates that the handling of the material during sampling and its exposure to oxygen initially activated ammonium uptake. It’s striking, though, that even after the first mineralization peaks, ranging between 50.5 ± 40.2 and 480 ± 150 mg N (kg DM day)^−1^, flattened out to a mean of 9.4 ± 1.9 mg N (kg DM day)^−1^, a large share of the mineralization rates observed exceeded by far those observed in soils. Braun et al. (2018) found gross mineralization rates for grassland soils of 4.07 ± 0.5 mg N (kg DM day)^−1^, whereas Habteselassie et al. (2006) measured 5.72 mg N (kg DM day)^−1^ in dairy-waste enriched agricultural soils. On day 110, a mean gross consumption of 35.7 ± 9.1 mg N (kg DM day)^−1^ was calculated for the aerated reactors, whereas gross production (mineralization) was about 3.7 ± 1.5 mg N (kg DM day)^−1^, a value in the range of the previously mentioned studies on soils. However, Elrys et al. (2021a) summarized the gross mineralization data in a global meta-analysis of soils and calculated a global average for gross nitrogen mineralization (GNM) of 8.63 ± 0.53 mg N (kg DM day)^−1^. Elrys et al. (2021a) also described total N as a one of the most important controls for gross nitrogen mineralization. Hence, the measured gross nitrogen mineralization rates during stable aerobic degradation of waste meets the global average better than individual soil data on specific ecosystems in comparable locations. This indicates that other factors, which were not taken into account in this study (e.g. total N, C:N ratio, humidity and pH), are of greater importance than site factors (e.g. climate). Combining the high gross consumption rates with very little gross mineralization resulted in a high net ammonium removal of − 32 ± 9.2 mg N (kg DM day)^−1^. The gross consumption rate remained quite stable until day 355 at 27 ± 6.2 mg N (kg DM day)^−1^, whereas net mineralization declined to − 10.6 ± 7.8 mg N (kg DM day)^−1^. The increasing share of gross production (mineralization) indicates maintenance metabolism of the microbiota, and that organic N used by the microbiota is fueling their C and energy metabolism, while excess N is mineralized and excreted, underlying the gross N mineralization process and causing positive net N mineralization rates (Mooshammer et al. 2014). At the end of the experiment (day 753) the gross consumption rates of the completely aerobic reactors were 6.8 ± 1 mg N (kg DM day)^−1^. Net mineralization rates decreased further to − 2.1 ± 1.2 mg N (kg DM day)^−1^. Investigating the aerobic–anaerobic reactors on day 753 the gross consumption rates declined slightly (but remained higher than for the aerobic group) to 20.1 ± 2 mg N (kg DM day)^−1^, whereas net mineralization remained stable at around − 10 ± 4.5 mg N (kg DM day)^−1^. The final measurement for the aerobic–anaerobic reactors is supported by two other observations: the steady increase in NH_4_-N concentration in leachate to 24 mg L^−1^ and the increase in microbial biomass (Fricko et al. 2021). At the experimental start, gross ammonium production rates were determined for the anaerobic reactors, which exceeded gross ammonium consumption, resulting in positive net mineralization (an ammonium release) of 23.8 ± 14.4 mg N (kg DM day)^−1^. This unexpected result might be explained by the implementation of the operation and the first sampling campaign. To ensure homogeneity as far as possible, the material for all reactors was piled, mixed and subsequently split up and transferred into the corresponding reactors. Then the material was stored inside the reactors themselves, until the reactors of each group were brought into service. For the anaerobic reactors a delay of 1 week resulted from this approach. During this period, the material was exposed to oxygen, but then quickly turned anaerobic again, leading to anaerobic degradation and consequently to the release of ammonium. In this stage, the effective sampling was performed.

#### Gross nitrification

**Fig. 2:**
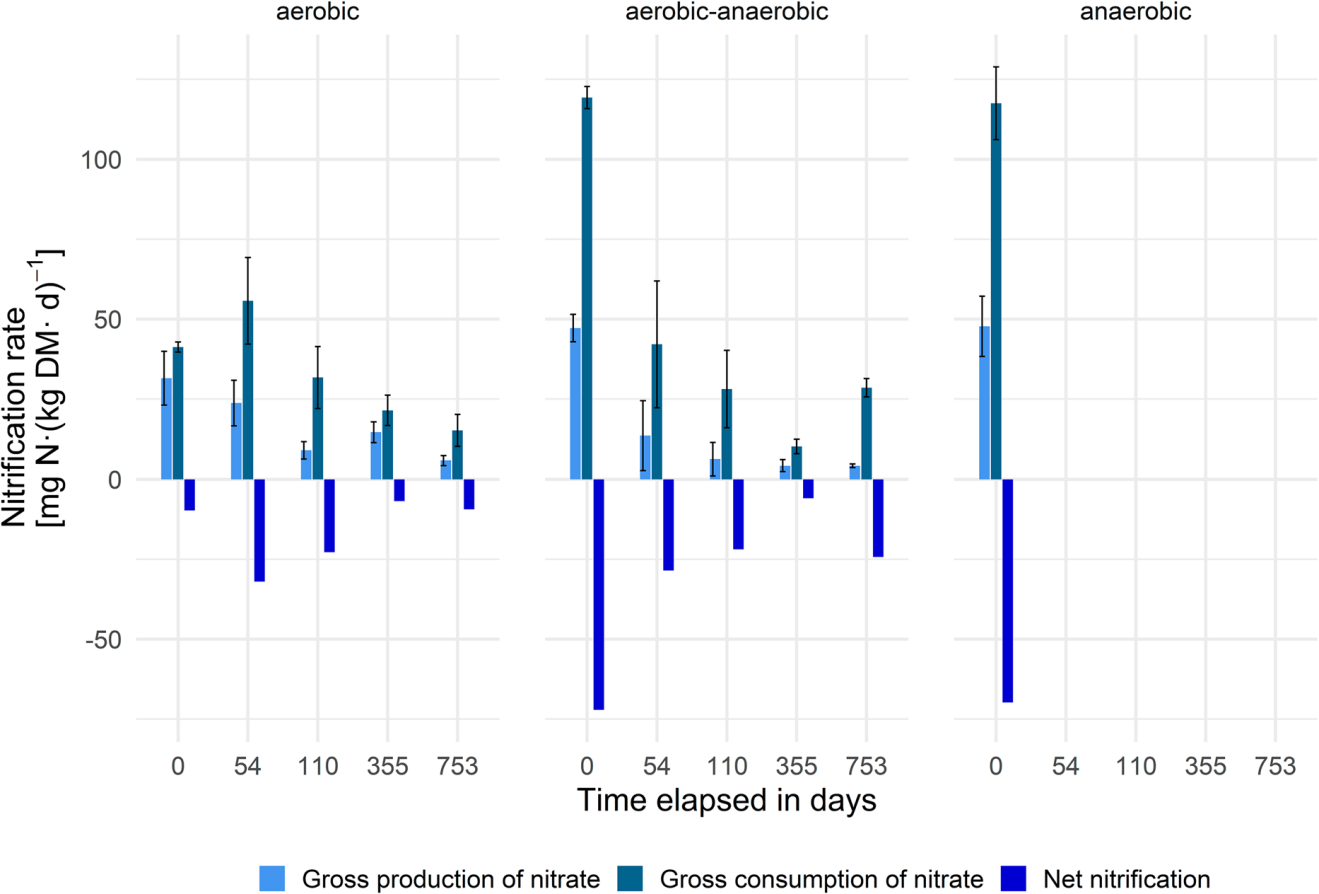
Nitrification rates per operation mode (n = 3) and experiment duration

Again, net nitrification is calculated by subtracting gross nitrate consumption from gross nitrate production (gross nitrification) rates. Generally, nitrification was expected to be more error prone in terms of measurement compared to measuring gross mineralization due to the required removal of the large excess of NH_4_-N using acid traps prior to converting nitrate to ammonium using Devarda’s reagent. As NH_4_-N exceeds NO_3_-N by far in anaerobic landfills, by an order of magnitude i.e. 5 × 10^3^ fold, even an incomplete removal of ammonium by “only” 99% would have caused serious impacts on the nitrification results. Moreover, given that nearly all measurements were performed as a “spike”, the rates display rather a potential for consumptive but likely less so for productive processes of nitrate, since consumptive processes are concentration dependent, while the production of nitrate via nitrifiers should be less impacted (and if so, negatively by feed-back inhibition) by nitrate concentration. Figure 2 gives an overview of the measured rates occurring over the experiment’s duration.

At day 0, the aerobic reactors displayed lower nitrate flux rates compared to the other groups. Again, the most probable explanation is the implementation of the reactors. The aerobic samples were incubated the following day after the transfer of the waste material inside the reactors, whereas the other groups were delayed by up to 1 week, and the reactors were not entirely sealed during this period. Once in contact with air, nitrifiers such as *Nitrosomonas* and *Nitrobacter* start to grow. Hence, the samples analyzed later achieved a better adapted microbial community than the first (aerobic) samples did. This consideration is supported by the fact that in the aerobic–anaerobic and the anaerobic 2 M KCl extracts for determining the amount of tracer to be added, small amounts of NO_3_-N (around 0.14 mg L^−1^) were detected. Thus, the aerobic measurement is considered more valid in terms of illustrating processes in a real MSW landfill. The subsequent sampling campaigns up to day 110 (355) showed a similar pattern for the aerobic as well as the aerobic–anaerobic groups. Once more, the measured rates are unusually high in comparison to individual soils, but the observation is in line with high gross mineralization rates since nitrification requires NH_4_-N as a precursor. Habteselassie et al. (2006) observed gross nitrification rates of 10.24 mg N (kg DM day)^−1^ in agricultural soils fertilized with dairy waste. Elrys et al. (2021b) calculated a global average of gross nitrification (GN) of 3.61 ± 0.21 mg N (kg DM day)^−1^, but mentioned that the average gross nitrification observed in croplands was significantly higher than in grasslands or forests. Considering the availability of NH_4_-N in these types of ecosystems, the similarity between waste material and cropland soil is obvious. Gross production (nitrification) rates derived from the experimental start at day 54 were around 18.7 ± 9 mg N (kg DM day)^−1^ and showed no significant difference between treatments, but a significant decline with time of incubation. 7.6 ± 3.9 mg N (kg DM day)^−1^ on day 110 resp. 15.8 ± 3.5 mg N (kg DM day)^−1^ (day 355) were calculated, whereas net nitrification declined constantly: before the start of aeration a net nitrification rate of − 30.3 ± 7.9 mg N (kg DM day)^−1^ was detected. Subsequently, net nitrification rates decreased to − 22.3 ± 8.8 mg N (kg DM day)^−1^ on day 110 and finally to − 6.4 ± 1.3 mg N (kg DM day)^−1^ on day 355. For the aerobic reactors, gross and net nitrification rates remained quite constant between day 355 and 753, with values at 5.8 ± 1.5 mg N (kg DM day)^−1^ resp. − 9.4 ± 3.8 mg N (kg DM day)^−1^, although these rates were not obtained from spiking but rather from labeling the nitrate pool. Again, the aerobic–anaerobic group showed a slight difference at the experimental end: The gross consumption of nitrate increased to 28.5 ± 2.9 mg N (kg DM day)^−1^, whereas the gross production of nitrate remained stable. Hence, net nitrification increased during the final campaign to 24.3 ± 2.4 mg N (kg DM day)^−1^. Considering the rates as a potential for nitrification, this increase is in line with the other observations, e.g. nitrate accumulation, and the already afore mentioned increase in microbial biomass.

**Fig. 3:**
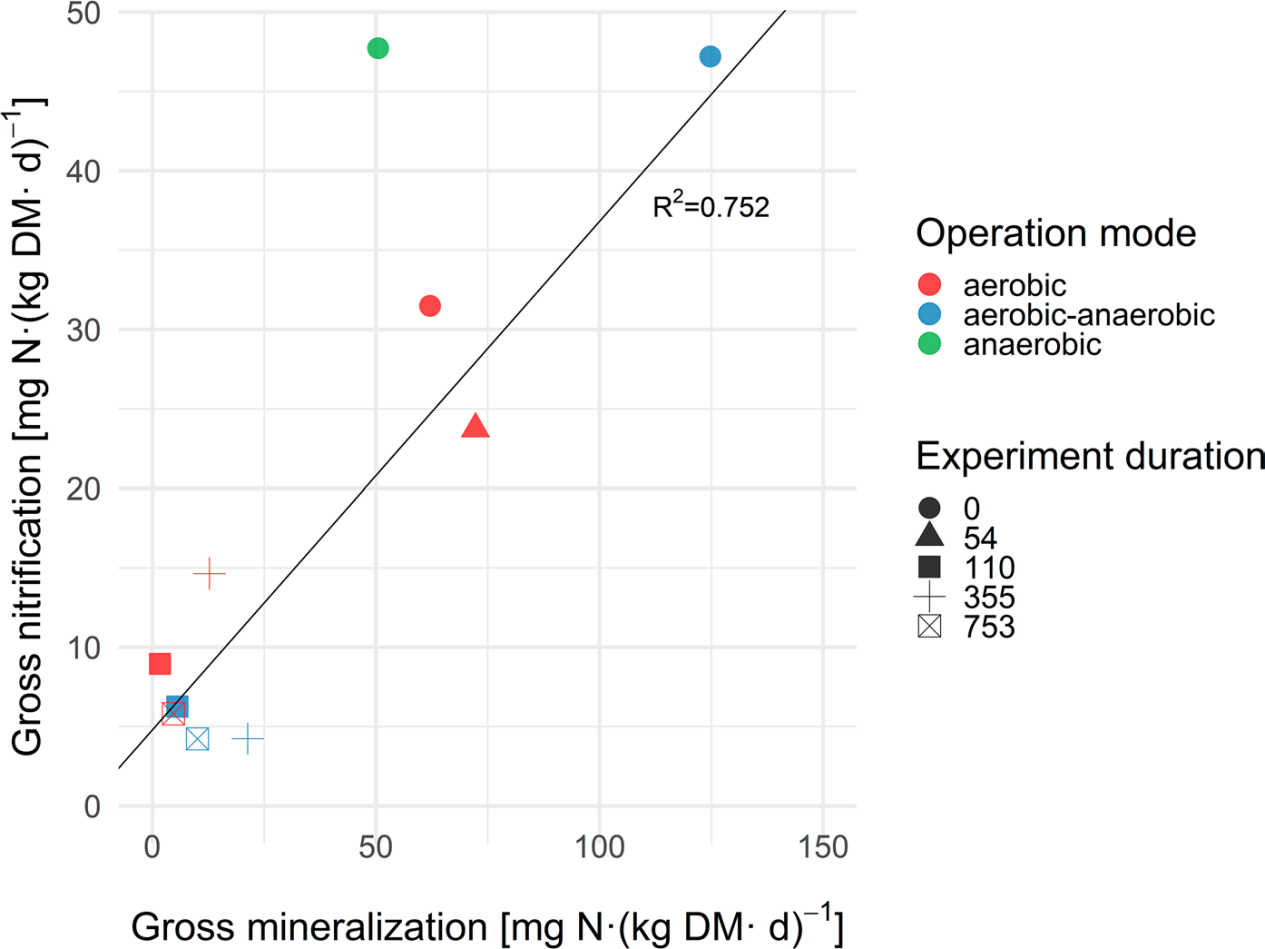
Gross nitrification rate displayed over gross mineralization per operation mode and experiment duration (n = 3)

Figure 3 summarizes the gross nitrification rates over the gross mineralization rates. As already discussed, only one measurement for gross mineralization for the anaerobic reactors on day 753 fulfilled the criteria of at%^15^N_t1_ exceeding at%^15^N_t2_. Without any statistical evaluation, a single measured rate tends to misrepresent the data. Hence, the anaerobic group on day 753 was not taken into account for the linear correlation. Similarly, the nitrification rates obtained for the aerobic–anaerobic and the anaerobic group at day 0 were considered to be overestimated due to the installation procedure and the resulting delay of sampling. Therefore they were also not considered for the correlation of gross nitrification rates and gross mineralization rates. In excluding the measurements for the aerobic–anaerobic and the anaerobic group on day 0 as well as the measurement of the anaerobic reactors at the end of the experiment (day 753), a relatively strong linear correlation was obtained.

Booth et al. (2005) described a linear dependency for log-transformed data on nitrification rates related to N mineralization rates in agricultural, grassland and woodland soils, but obtained the best fit for the untransformed data using a power function with decreasing proportions for N being nitrified when mineralization rates increased. This mathematical approach could also fit the data displayed in Fig. 3, but due to the lack of additional data points there was no possibility to evaluate that. The described decline in mineralization and nitrification rates (potential) from day 110 onwards is also obvious in Fig. 3. Further controls of nitrification in soils are the extractable amount of NH_4_-N and the pH (Booth et al. 2005). For NH_4_-N this also seems true for waste material — the observed decline to (almost) zero in NH_4_-N concentrations in leachate happened within 2 weeks after the start of aeration. Mineralization and nitrification rates measured at the subsequent sampling event (day 110) were by far lower than those observed during the initial anaerobic phase. The pH of waste material does not exhibit such a strong variation as it does in soils. For the anaerobic reactors, a pH of 7.5–7.8 was measured, while slightly lower values were obtained for the aerated reactors (6.9–7.5). A correlation between pH and mineralization rates was not observed.

#### ^15^N_2_O

The results for the concentration and isotopic composition of N_2_O formed during incubation were validated utilizing the data obtained from the non-labeled samples. All samples, except the sample retrieved at day 110, mirrored the background concentration of N_2_O in the atmosphere (currently 331 ppb or 0.33 ppm), indicating that net N_2_O production occurred only around the time of the start of aeration. After excluding these measurements, the remaining 60 measurements were summarized to 0.30 ± 0.01 ppm and with an isotopic composition of 0.41 ± 0.03at%^15^N, which is close to natural abundance around 0.37at%^15^N. For the majority of the measurements, background concentrations and isotopic compositions were also measured after 4 h of incubation, supporting the assumption of 4 h being a well-chosen time point for rate calculations. N_2_O is an undesirable by-product of nitrification and of incomplete denitrification and therefore its production can be stimulated by increased ammonium and nitrate production as substrates for nitrifiers and denitrifiers. Hence, the enrichment of ^15^N_2_O measured cannot exceed the share of at%^15^N added through any of the tracer substances (more or less 10at%^15^N), a requirement which is clearly met by all samples, but should exceed those of background N_2_O to enable exact rate determinations. To comply with the requirements of the isotope pool dilution the results were restricted to those in which at%^15^N_t2_ was lower than at%^15^N_t1_. All measurements met these requirements.

**Fig. 4:**
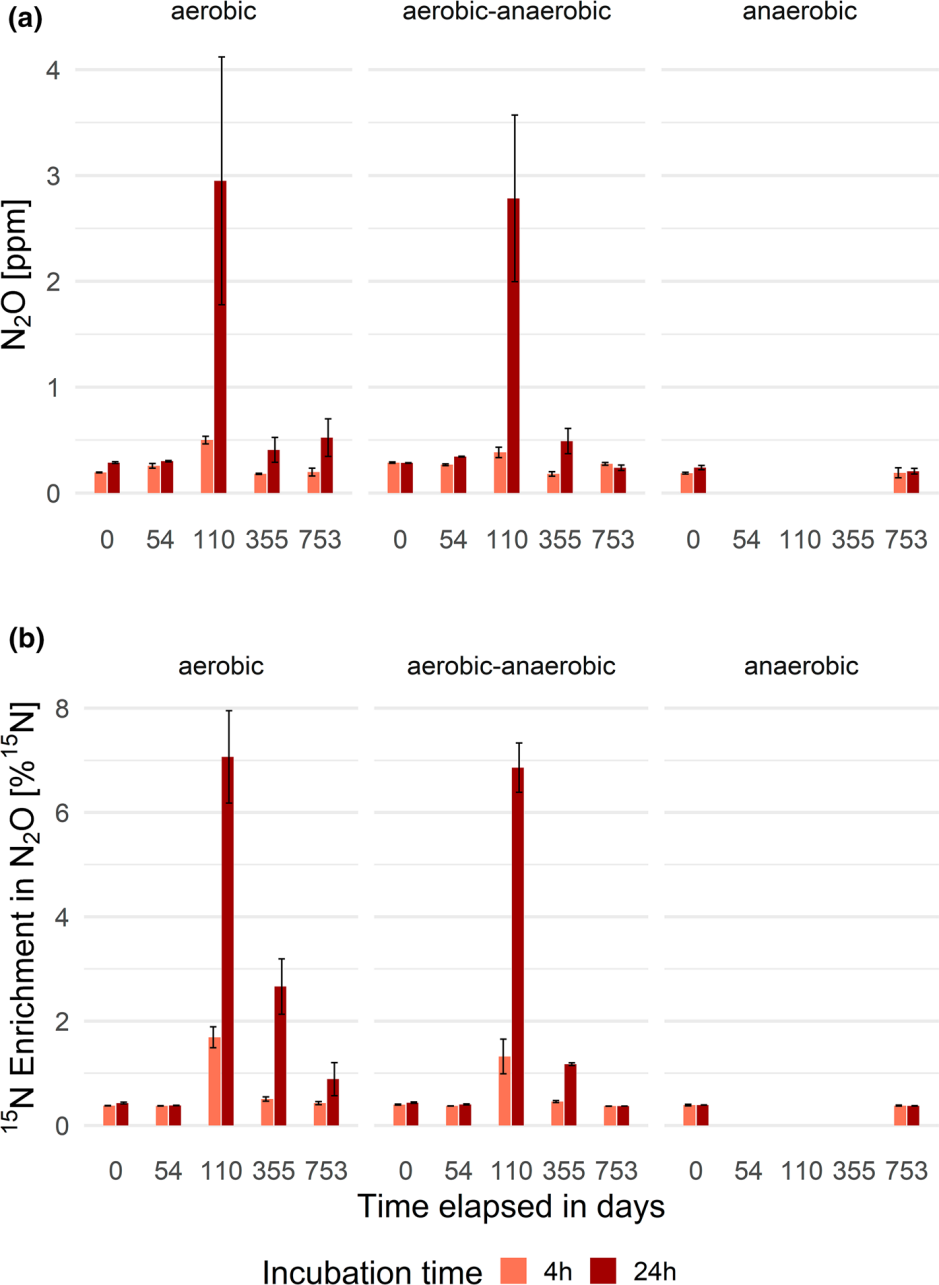
**a** ^15^N_2_O concentrations [ppm] and **b** corresponding enrichment of ^15^N in N_2_O [%] occurring during mineralization-incubation per operation mode (n = 3) and experiment duration

**Fig. 5:**
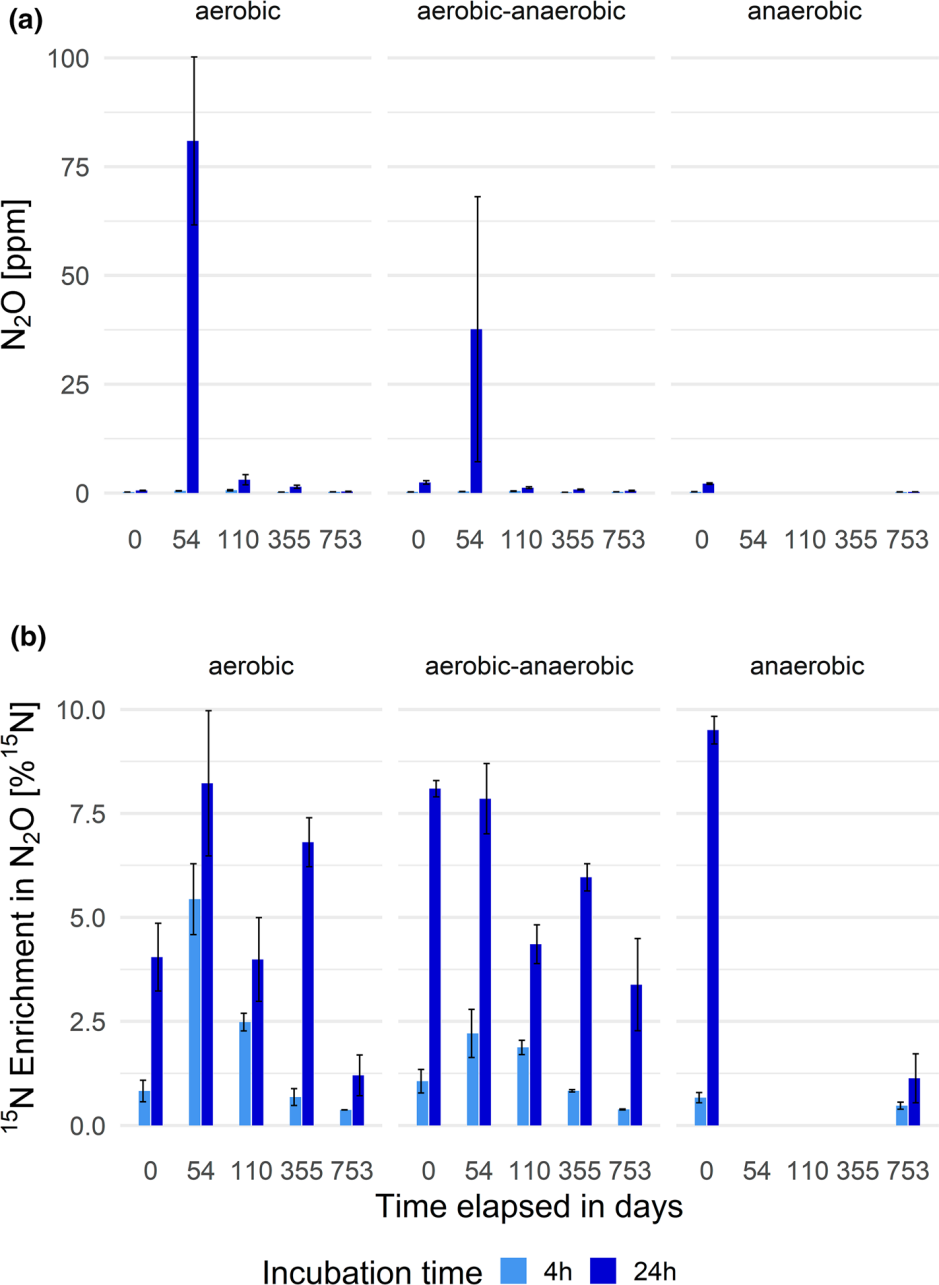
**a** ^15^N_2_O concentrations [ppm] and **b** corresponding enrichment of ^15^N in N_2_O [%] occurring during nitrification-incubation per operation mode (n = 3) and experiment duration

In Fig. 4 the concentrations and corresponding enrichments of N_2_O formed during mineralization-incubations are summarized; in Fig. 5 those during nitrification-incubations are likewise summarized.

Only five measurements deviate significantly from the background concentration and composition of N_2_O, namely those determined by oxygen availability. N_2_O production after ^15^NH_4_Cl tracer addition (or spike) is a consequence of mineralization and nitrification (or coupled nitrification–denitrification within the 24 h measurement window) in the aerobic state. Specifically, this is valid for the aerobic group on days 110, 355 and 753 as well as for the aerobic–anaerobic reactors on day 110 and 355, where net N_2_O production clearly happened. The peak in both concentration (3–4 ppm) and ^15^N enrichment of N_2_O reached 70% of the maximum enrichment on day 110 in both groups and might be explained by the slow adaption of the microbiota to aerobic conditions (slow growth of *Nitrobacter*). As adjustment to the treatment conditions increased, N_2_O production from ammonium decreased, as indicated by lower net N_2_O accumulations of 0.1–0.5 ppm and lower ^15^N enrichments of < 10% of the maximum. The pattern for ^15^N enrichment fitted the measured concentrations well.

Focusing on day 0, again deviations between the aerobic group and the others were visible. This was already discussed for nitrification rates and is most probably the result of the installation and implementation of the reactors. The peak in N_2_O formation in nitrate amendments on day 54 seems contradictory, but the corresponding nitrification rates indicate a high potential for nitrification. Hence, the added nitrate spike was metabolized, but not completely denitrified, resulting in an enormous release of up to 75 ppm N_2_O on day 54, with high ^15^N enrichments, reaching 80% of maximal achievable ^15^N enrichments. On day 110 resp. 355 the microbiota were capable of handling the spike (due to the ongoing aeration), though net N_2_O formation declined to 1–5 ppm. However, this was still roughly ten-times higher than in the ammonium amendments. As aeration or anaerobiosis was continued or triggered, net N_2_O formation further declined but ^15^N enrichments remained higher than in the nitrifier assays with ^15^N ammonium amendments, at around 10–40% of the maximal enrichments achievable. In contrast to N_2_O formed during the mineralization-incubation (nitrification), strong ^15^N_2_O enrichment was detected in nitrification-incubation samples with only little N_2_O production in terms of concentration, indicating other metabolic activity such as complete denitrification.

After both incubations N_2_O formation was detected. Nonetheless, the concentration (and isotopic enrichment) of N_2_O due to incomplete denitrification exceeded the concentration (and isotopic enrichment) of N_2_O formed by incomplete nitrification by a factor of 20 (and 10), indicating the clear dominance of denitrifier-N_2_O over nitrifier-N_2_O source processes.

### δ^15^N natural abundance and bulk concentrations

Table 1 gives an overview of the concentrations of TN, NH_4_-N, NO_3_-N and their specific isotopic composition for each operational group and sampling event.

**Table 1:** Concentrations and δ-values of total N and N-species per operational group (n = 3) and experiment duration

Experiment duration	Operation mode	TN	NH_4_-N	NO_3_-N	δ^15^N TN	δ^15^N NH_4_-N	δ^15^N NO_3_-N
Days	**–**	mg kg DM^−1^	mg kg DM^−1^	mg kg DM^−1^	‰	‰	‰
0	Aerobic	5128 ± 242	927 ± 87	26.6 ± 1.9	5.5 ± 0.3	11.8 ± 1.3	47.4 ± 13.7
0	Aerobic–anaerobic	5136 ± 103	1040 ± 70	28.4 ± 2.1	5.6 ± 0.1	17.2 ± 3.1	30.7 ± 3
0	Anaerobic	4598 ± 485	1035 ± 112	27.2 ± 2.3	5.6 ± 0.4	15 ± 3.7	32.9 ± 6.8
54	Aerobic	5112 ± 96	923 ± 52	11.7 ± 0.1	5.6 ± 0.4	7.8 ± 0.3	22.8 ± 2.6
54	Aerobic–anaerobic	5489 ± 76	1037 ± 23	12.6 ± 0.2	5.5 ± 0.6	12.1 ± 2.4	19.9 ± 1.5
110	Aerobic	4604 ± 108	15.9 ± 0.7	25.6 ± 10.3	3.2 ± 0.5	41.7 ± 12.9	57.3 ± 12.9
110	Aerobic–anaerobic	5060 ± 8	13.7 ± 0.6	20.9 ± 4.3	3.8 ± 0.2	19.8 ± 2.3	63.8 ± 2.8
355	Aerobic	4234 ± 180	20.8 ± 0.6	13.4 ± 4.6	4.1 ± 0.3	122 ± 9	504 ± 161
355	Aerobic–anaerobic	4740 ± 53	21.8 ± 0.8	41.8 ± 4.8	4.1 ± 0.4	207 ± 47	746 ± 144
753	Aerobic	4899 ± 46	11.1 ± 0.7	53.6 ± 22.5	11 ± 4.8	14.2 ± 2.4	202 ± 62
753	Aerobic–anaerobic	5062 ± 91	151.5 ± 9.5	6.1 ± 0.3	11.5 ± 5.8	9.3 ± 0.1	607 ± 223
753	Anaerobic	4790 ± 328	880.6 ± 13.5	6.2 ± 0.2	5 ± 0.2	7.2 ± 0.2	373 ± 114

Looking at the data for natural ^15^N abundance (Table 1), δ^15^N in TN started from 5.6 ± 0.3‰ at day 0 for all reactors. Until day 54, no significant changes occurred. Subsequent to the start of aeration (day 110), δ^15^N in TN decreased to 3.5 ± 0.4‰ in aerobic and aerobic–anaerobic treatments. After a year of operation, on day 355, δ^15^N in TN had increased slightly to 4.1 ± 0.4‰. Regardless of the operation mode, for both, completely aerobic and aerobic–anaerobic groups at the end of the experiment (day 753), the highest δ^15^N values with the strongest variations were observed, with an average of 11.3 ± 5.3‰, indicating isotope fractionating nitrogen losses. These can occur through hydrological losses of nitrate or gaseous losses of ammonia, NO, N_2_O or N_2_, triggered by high pH (ammonia) or by nitrifiers (NO, N_2_O) and denitrifiers (NO, N_2_O, N_2_), which all exert strong isotope effects of their residual substrate (becoming ^15^N enriched) and their cumulative product (becoming ^15^N depleted) (Denk et al. 2017; Högberg 1997). The completely anaerobic reactors, on the other hand, remained quite stable, with δ^15^N in TN of 5.0 ± 0.2‰, indicating relatively minor reactive nitrogen losses since nitrification, the key process to trigger strongly ^15^N fractionating losses, was suppressed. The increase in δ^15^N in TN due to aeration matches the results of Lynch et al. (2006), who investigated the changes in nitrogen content and natural ^15^N abundance (δ^15^N) in corn silage compost over 9 months and observed an increase of δ^15^N from 0.3 ± 1.3 to 8.2 ± 0.4‰ in combination with a loss of 28.4% from the initial nitrogen content. However, the trend towards more uniform values for δ^15^N, as observed by Lynch, was not found here. A possible explanation is the different handling of the compost in comparison to the in-situ aeration of waste: compost is thoroughly mixed and relatively homogenous, whereas waste material remains static and highly heterogeneous.

δ^15^N of NH_4_-N was also very similar between the operational groups at the start of the experiment, at around 14.67 ± 2.70‰, but tended to vary more strongly with increasing experimental duration. The isotopic composition of ammonium is controlled by the fraction and extent of isotope fractionation by mineralization (ammonification, deamination), causing ammonium to be ^15^N depleted relative to TN and subsequent ammonium consumption by nitrifiers, in turn causing ammonium to become ^15^N enriched. After a minor decrease at day 54 to 9.95 ± 1.35‰, an increase after the start of aeration (day 110) to 30.75 ± 7.60‰ followed, indicating a larger fraction of ammonium to become nitrified. This development reached its peak after 1 year of operation on day 355, with a δ^15^N value for NH_4_-N of 165 ± 28‰, indicating almost complete nitrification of the ammonium produced during mineralization. Until then no differences between the groups were detected. For the final sampling on day 753 the ammonium δ^15^N value for the aerobic group had decreased again to 14.2 ± 2.4‰. The aerobic–anaerobic reactors had declined even further to 9.3 ± 0.1. However, the lowest value − 7.2 ± 0.2—was displayed for the completely anaerobic group, indicating complete inhibition of nitrification under anoxic conditions.

Considering the δ^15^N values of NO_3_-N, all reactors started at around 37 ± 7.83‰, the highest isotopic enrichment of all three measured nitrogen species. Since nitrifiers produce nitrate with a ^15^N depleted isotope signature (roughly 30–40‰ lower than its substrate ammonium), the data clearly indicate that a large proportion of nitrate was consumed by denitrifiers. This causes nitrate to become increasingly ^15^N enriched the larger the fraction of nitrate denitrified becomes, and the gaseous products of denitrification are ^15^N depleted by up to 15–35‰ (Denk et al. 2017), causing an overall system ^15^N enrichment as found in TN in (partially) aerobic incubations, where nitrifiers produce ample substrate for denitrifiers. Similarly to δ^15^N NH_4_-N, a decrease to 21.35 ± 2.05‰ followed on day 54. From day 110 onwards, a constant increase was observed, first to 60.55 ± 7.85‰ and subsequently to 625 ± 153‰ on day 355. At the end of the experiment, the aerobic reactors experienced a decline in δ^15^N NO_3_-N to 202 ± 62‰, whereas the aerobic–anaerobic group remained at 607 ± 223‰, as high as before aeration was terminated. Interestingly, elevated δ^15^N values for nitrate (373 ± 114‰) for the completely anaerobic reactors were also measured, highlighting that the little nitrate produced or leaked in the anaerobic system were also almost completely denitrified. With the start of aeration (day 54 onwards), oxygen became easily available and hence nitrification was stimulated. Consumptive processes prevail over the production of nitrate, but in the long term nitrate concentration increased in the aerobic environment. Hence, the increase in isotopic fractionation on day 110 resp. 355 was the result of high nitrate turnover since microbiota tend to assimilate or dissimilate ^14^N preferably to ^15^N species and therefore enrich ^15^N in the remaining substrate. This cycle is broken when nitrate accumulates, as in the aerobic reactors on day 753, where the constant influx of ^14^NO_3_-N reduced the isotopic fractionation significantly while denitrification under suboxic to oxic conditions declined. For the aerobic–anaerobic group, the termination of aeration on day 355 blocked the nitrification pathway but stimulated denitrification, thus δ^15^N NO_3_-N did not change during the following period. The elevated isotopic enrichment of nitrate of the completely anaerobic group might be a result of the intrusion of air into the system, with O_2_ intrusion allowing low levels of nitrification, which should have been excluded for these reactors as well.

## Conclusion

The measured gross mineralization and nitrification rates calculated reflect the huge potential for nitrogen transformations within the waste material. However, as only the fraction ≤ 4 mm was considered for the isotope pool dilution measurements, the gross reaction rates tend to be overestimated. Mean gross nitrogen mineralization (production of NH_4_) rates of 158 ± 68 mg N (kg DM day)^−1^ were observed in the anaerobic state at the beginning of the experiment, which is 20–100 times higher than those reported by Braun et al. (2018) and Booth et al. (2005) for cropland, grassland and forest soils. Even during aeration gross mineralization rates [9.2 ± 1.8 mg N (kg DM day)^−1^] remained approximately twice as high as compared to soils. Mean gross nitrification (production of NO_3_) rates were significantly lower than gross nitrogen mineralization rates at 32.7 ± 8 mg N (kg DM day)^−1^, but still three times higher than those measured by Habteselassie et al. (2006) in agricultural soils and then synthesized from the available literature by Booth et al. (2005) for cropland, grassland and forest soils. As a consequence of prolonged aeration and substrate (NH_4_) depletion, gross nitrification rates declined to 8.0 ± 2.9 mg N (kg DM day)^−1^. A positive correlation between gross nitrogen mineralization and gross nitrification was obtained (Fig. 3), indicating their dependency on overall biodegradation rates of the waste and that, provided there is sufficient oxygenation, the supply of ammonium is the limiting factor for nitrifier activity. As discussed previously, waste material is far more heterogeneous than any soil and the concentrations of NH_4_-N exceeded the concentrations of NO_3_-N by orders of magnitude under anaerobic operation, resulting in methodological challenges and high variability in the rates measured, and large pool sizes relative to “small” fluxes may have further constrained the application of the isotope pool dilution method.

Additionally, measurements regarding ^15^N_2_O formation during the incubation experiments confirm that the main source of N_2_O is incomplete denitrification (max. 75 ppm, high ^15^N_2_O enrichments), not (incomplete) nitrification (3–4 ppm, lower ^15^N_2_O enrichments). δ^15^N TN showed a significant increase during aeration, starting quite uniformly at 5.6 ± 0.3‰ ultimately reaching 11.3 ± 5.3‰ at the end of the whole experiment.

## Supplementary Information

Below is the link to the electronic supplementary material.

Supplementary file1 (DOCX 618kb)
