# Supplementary material for: Applying the 15N labelling technique to material derived from a landfill simulation experiment to understand nitrogen cycle processes under aerobic and anaerobic conditions
Source: Biodegradation. 2022 Oct 11;33(6):557–73. doi: 10.1007/s10532-022-10000-7 (PMC9581851; doi:10.1007/s10532-022-10000-7)
Supplement: Supplementary file 1 — Supplementary file1 (DOCX 618kb) [file 10532_2022_10000_MOESM1_ESM.docx]

# Supplementary Information to: Applying the ^15^N labelling technique to material derived from a landfill simulation experiment to understand nitrogen cycle processes under aerobic and anaerobic conditions

Nora Fricko*, Wolfgang Wanek, Johann Fellner


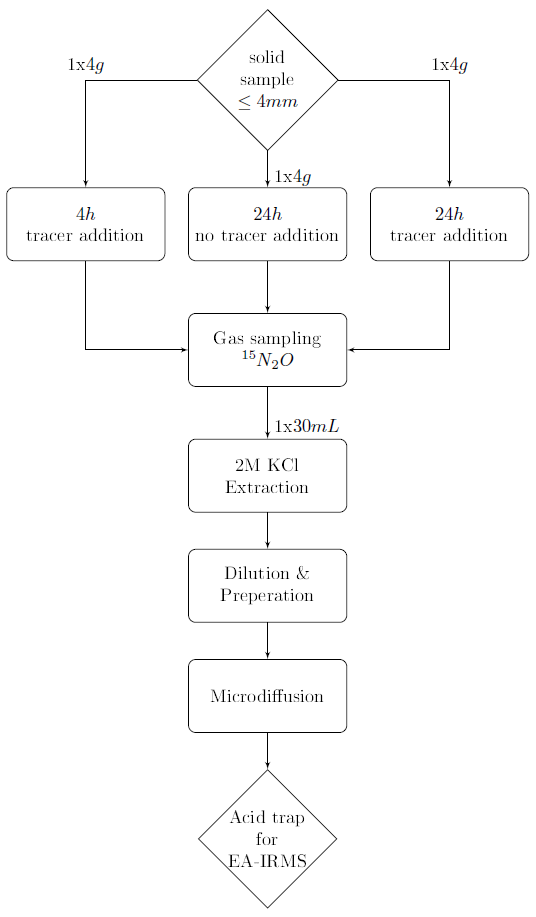


Figure S1 Incubation procedure for each solid sample derived from the LSRs (for NH_4_-N as well as NO_3_-N)

Table S1 Tracer solutions for NH_4_-N incubation and applied dilution (microdiffusion)

| **Experiment duration** | **Operation mode** | **NH_4_-N** | **type** | **^15^NH_4_Cl** | **NH_4_Cl** | **Dilution** |  |
| --- | --- | --- | --- | --- | --- | --- | --- |
|  |  |  |  |  |  |  |  |
| **d** | **-** | **mg·L^-1^** | **-** | **mg·10mL^-1^** | **mg·10mL^-1^** | **-** |  |
| 0 | aerobic | 103.3 | pool | 15.030 | - | 100 |  |
| 0 | aerobic-anaerobic | 100.1 | pool | 13.051 | - | 100 |  |
| 0 | anaerobic | 113.0 | pool | 16.308 | - | 100 |  |
| 54 | aerobic | 70.3 | pool | 10.352 | - | 100 |  |
| 54 | aerobic-anaerobic | 84.9 | pool | 12.032 | - | 100 |  |
| 110 | aerobic | 1.68 | spike | 0.987 | 8.283 | 10 |  |
| 110 | aerobic-anaerobic | 0.65 | spike | 0.976 | 8.251 | 10 |  |
| 355 | aerobic | 0.40 | spike | 0.938^a^ | 8.203^a^ | 5 |  |
| 355 | aerobic-anaerobic | 1.53 | spike | 0.938^a^ | 8.203^a^ | 5 |  |
| 753 | aerobic | 0.65 | spike | 0.953 | 8.419 | 5 |  |
| 753 | aerobic-anaerobic | 11.1 | pool | 1.616 | - | 10 |  |
| 753 | anaerobic | 70.5 | pool | 10.195 | - | 100 |  |

^a^ prepared as one solution (20mL)

Table S2 Tracer solutions for NO_3_-N incubation and applied dilution (microdiffusion)

| **Experiment duration** | **Operation mode** | **NO_3_-N** | **type** | **K^15^NO_3_** | **KNO_3_** | **Dilution** |  |
| --- | --- | --- | --- | --- | --- | --- | --- |
|  |  |  |  |  |  |  |  |
| **d** | **-** | **mg·L^-1^** | **-** | **mg·10mL^-1^** | **mg·10mL^-1^** | **-** |  |
| 0 | aerobic | 0.02 | spike | 1.294 | 13.811 | 25 |  |
| 0 | aerobic-anaerobic | 0.03 | spike | 1.787 | 13.249 | 25 |  |
| 0 | anaerobic | 0.25 | spike | 1.712 | 13.317 | 25 |  |
| 54 | aerobic | 0.06 | spike | 1.761 | 13.376 | 10 |  |
| 54 | aerobic-anaerobic | 0.14 | spike | 1.510 | 13.492 | 10 |  |
| 110 | aerobic | 0.56 | spike | 1.728 | 13.546 | 10 |  |
| 110 | aerobic-anaerobic | 0.53 | spike | 1.628 | 13.358 | 10 |  |
| 355 | aerobic | 0.21 | spike | 1.534^a^ | 13.626^a^ | 5 |  |
| 355 | aerobic-anaerobic | 0.75 | spike | 1.534^a^ | 13.626^a^ | 5 |  |
| 753 | aerobic | 4.3 | pool | 1,151 | - | 5 |  |
| 753 | aerobic-anaerobic | 0.32 | spike | 1.694 | 13.628 | 5 |  |
| 753 | anaerobic | 0.17 | spike | 1.559 | 13.852 | 5 |  |

^a^ prepared as one solution (20mL)


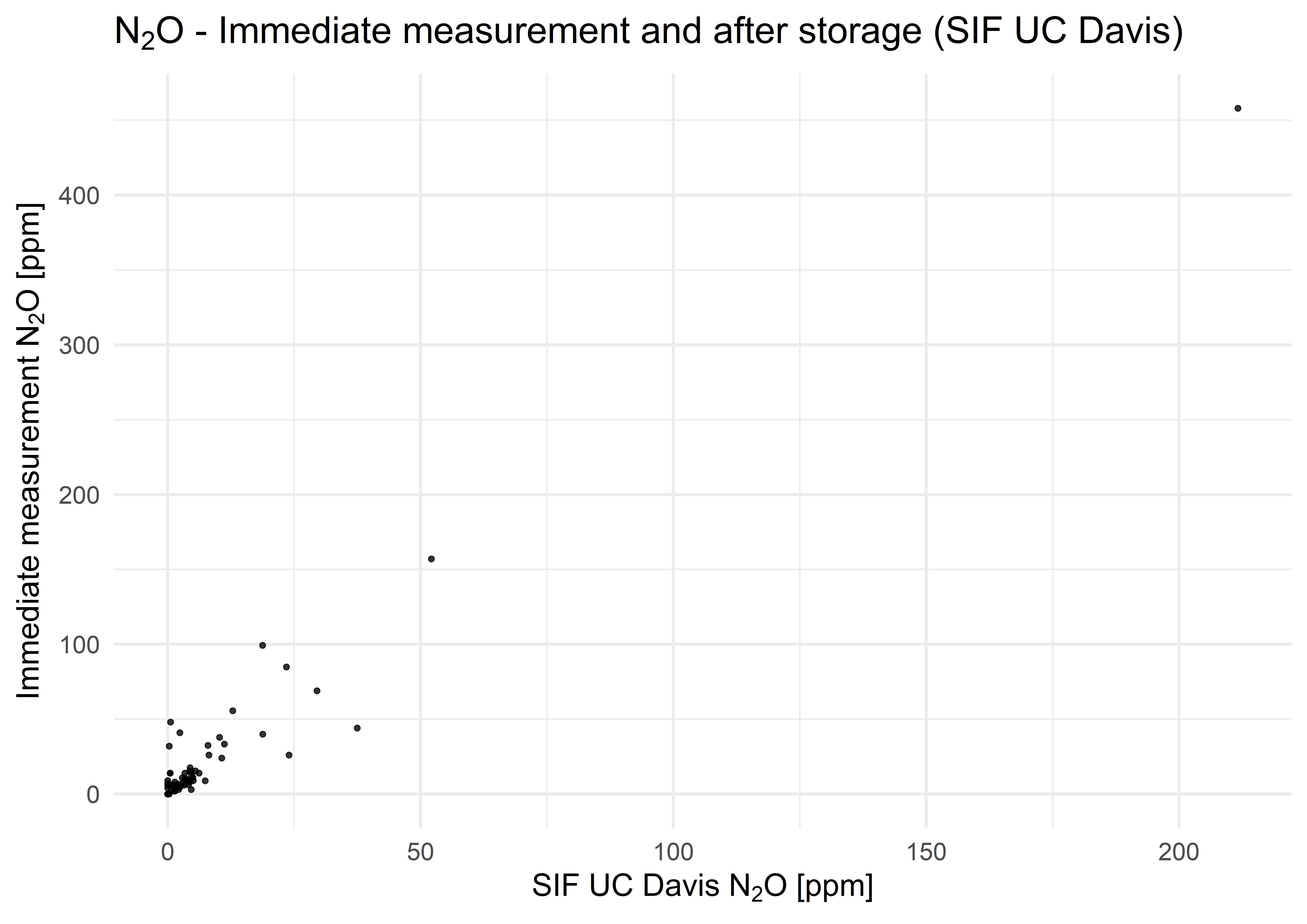


Figure S2 Comparison of N_2_O measurements immediately conducted and conducted at UC Davis, California (n=102)

Table S3 Different spike concentrations tested on day 355 (resp. 358, n=6)

| **Spike** | | **^15^NH_4_Cl** | **NH_4_Cl** | **Dilution** | **K^15^NO_3_** | **KNO_3_** | **Dilution** |  |
| --- | --- | --- | --- | --- | --- | --- | --- | --- |
|  |  |  |  |  |  |  |  |  |
|  | **mmol·10mL^-1^** | **mg·20mL^-1^** | **mg·20mL^-1^** | **-** | **mg·20mL^-1^** | **mg·20mL^-1^** | **-** |  |
| **standard** | 0,150 | 1,875 | 16,405 | 5 | 3,068 | 27,251 | 5 |  |
| **0.5** | 0,075 | 1,192 | 8,492 | 2 | 1,335 | 13,612 | 2 |  |
| **1.5** | 0,225 | 2,858 | 24,609 | 5 | 4,669 | 40,184 | 5 |  |
| **2.0** | 0,300 | 3,601 | 32,372 | 5 | 6,107 | 54,996 | 5 |  |


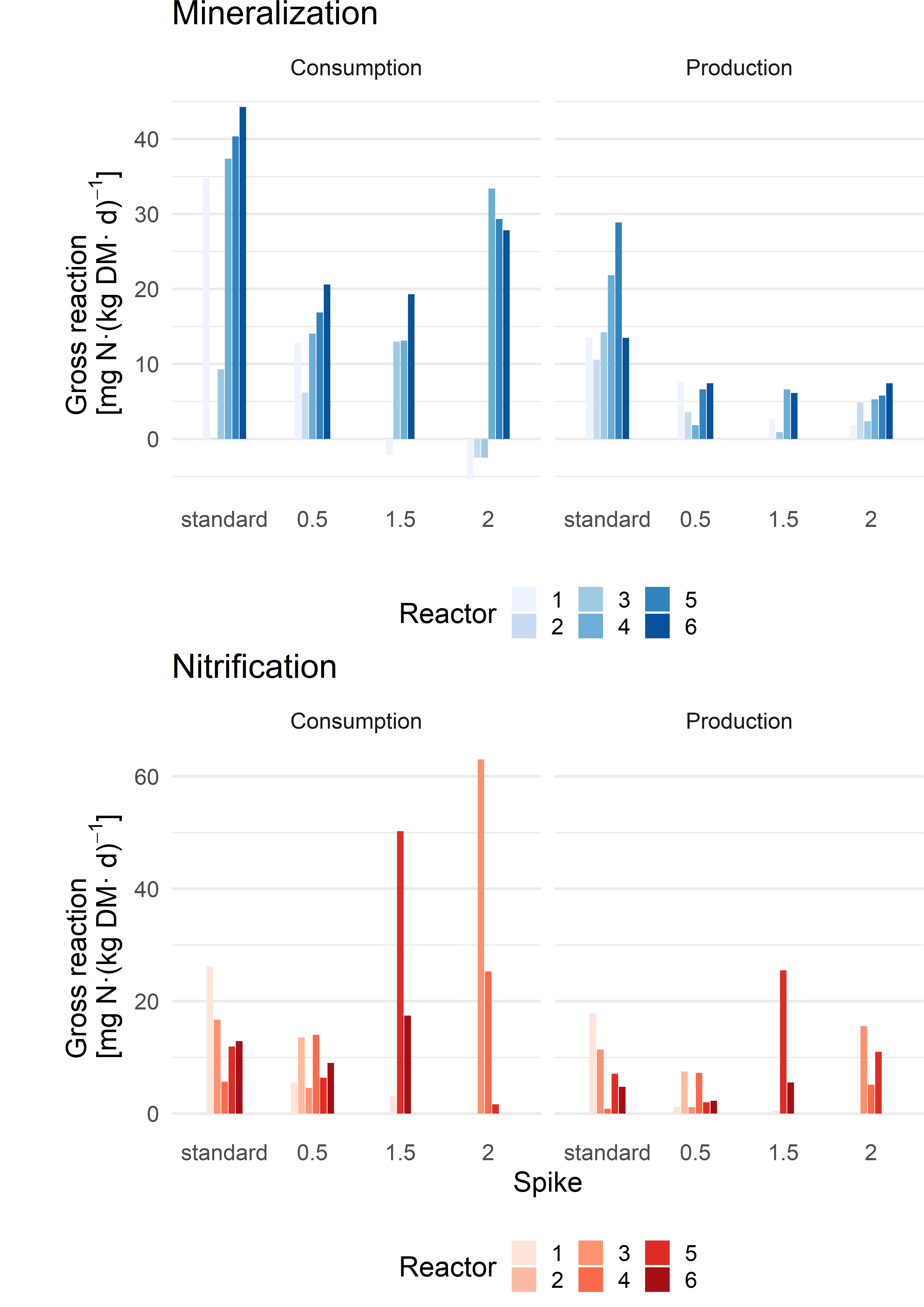


Figure S3 Gross reaction rates for mineralization and nitrification applying different spike concentrations during solid sampling on day 355 (resp. 358, n=6)


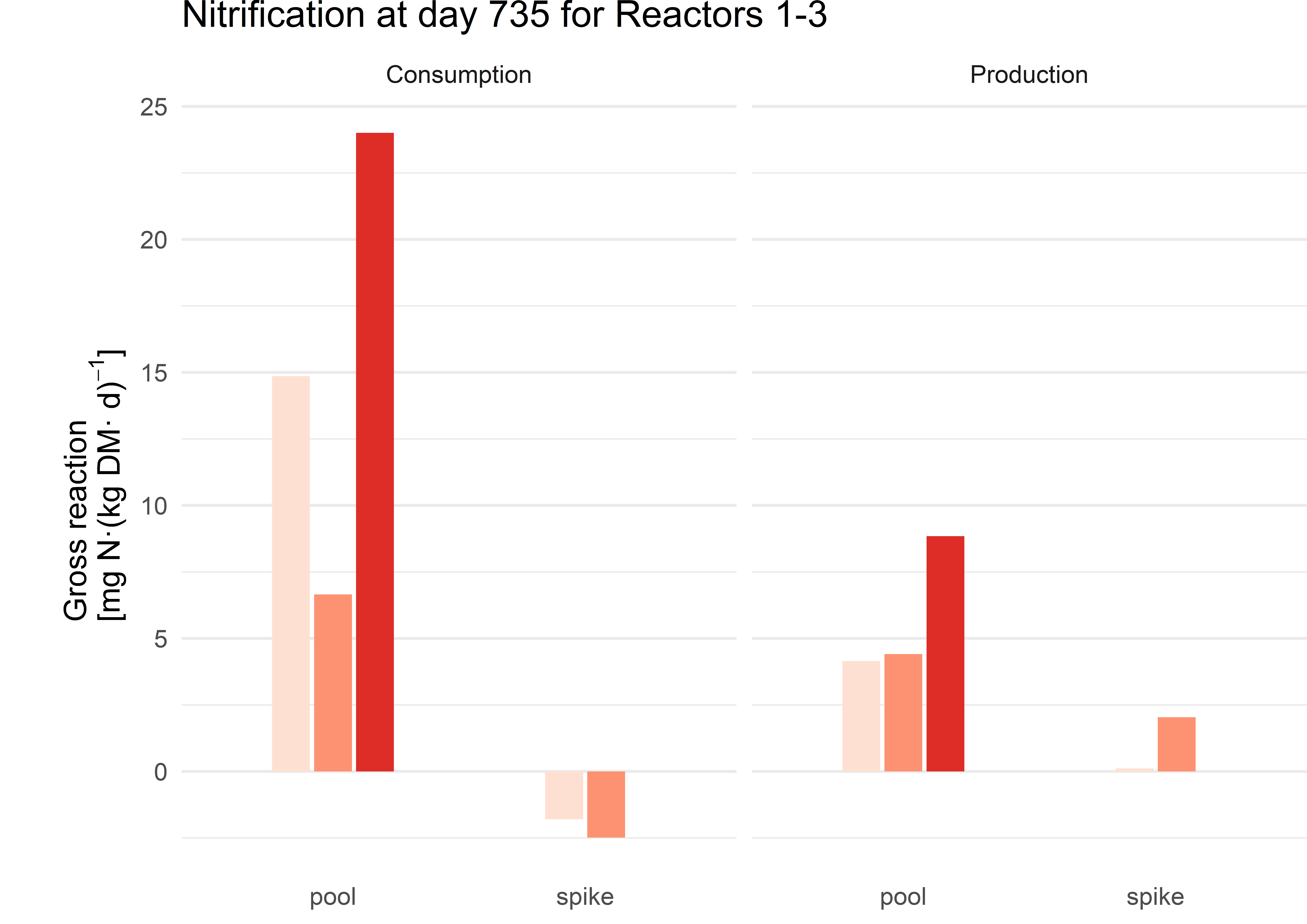


Figure S4 Gross nitrification rates obtained either by spiking with 0.15mmol·10mL^-1^ (“spike”) or by 10% tracer addition (“pool”) at the end of the experiment (day 753) for the aerated reactors (n=3)

Table S4 Significance level p for mineralization and nitrification rates compared for different sampling events for each operation mode (p < 0.05)

| experiment date | ammonium | | | | nitrate | | | |
| --- | --- | --- | --- | --- | --- | --- | --- | --- |
|  | aerobic | | aerobic-anaerobic | | aerobic | | aerobic-anaerobic | |
|  | production | consumption | production | consumption | production | consumption | production | consumption |
| 54-0 | 0.9984 | 0.7887 | 0.0750 | 0.9431 | 0.8269 | 0.8319 | 0.0065* | 0.0015* |
| 110-0 | 0.5786 | 0.9997 | 0.8367 | 0.9371 | 0.0873 | 0.9549 | 0.0008* | 0.0002* |
| 355-0 | 0.6656 | 0.9908 | 0.8928 | 0.9492 | 0.3134 | 0.7012 | 0.0005* | 0.0000* |
| 753-0 | 0.5447 | 0.9859 | 0.8538 | 0.9358 | 0.0488* | 0.4012 | 0.0005* | 0.0002* |
| 110-54 | 0.3685 | 0.6834 | 0.0086* | 0.4971 | 0.2583 | 0.3777 | 0.8281 | 0.8137 |
| 355-54 | 0.4181 | 0.4580 | 0.0106* | 0.5250 | 0.7364 | 0.1938 | 0.6791 | 0.1828 |
| 753-54 | 0.3125 | 0.4286 | 0.0091* | 0.4944 | 0.1381 | 0.0660 | 0.6783 | 0.8280 |
| 355-110 | 0.9977 | 0.9991 | 0.9999 | 1.0000 | 0.9327 | 0.9416 | 0.9972 | 0.5588 |
| 753-110 | 1.0000 | 0.9981 | 1.0000 | 1.0000 | 0.9876 | 0.6836 | 0.9972 | 1.0000 |
| 753-355 | 0.9990 | 1.0000 | 1.0000 | 1.0000 | 0.7551 | 0.9898 | 1.0000 | 0.5402 |

*significant

Table S5 Significance level p for mineralization and nitrification rates compared for different operation modes at a specific sampling event (p < 0.05)

|  |  | ammonium | | | nitrate | | |
| --- | --- | --- | --- | --- | --- | --- | --- |
|  | experiment  date | aerobic- aerobic-anaerobic | aerobic- anaerobic | aerobic-anaerobic- anaerobic | aerobic- aerobic-anaerobic | aerobic- anaerobic | aerobic-anaerobic- anaerobic |
| production | 0 | 0.7137 | 0.9871 | 0.6346 | 0.4186 | 0.3985 | 0.9986 |
|  | 54 | 0.0569 | - | - | 0.4686 | - | - |
|  | 110 | 0.1733 | - | - | 0.6687 | - | - |
|  | 355 | 0.1356 | - | - | 0.0539 | - | - |
|  | 753 | 0.1133 | - | - | 0.3873 | - | - |
| consumption | 0 | 0.7434 | 0.9852 | 0.6581 | 0.0029* | 0.0032* | 0.9845 |
|  | 54 | 0.5147 | - | - | 0.5943 | - | - |
|  | 110 | 0.1048 | - | - | 0.8279 | - | - |
|  | 355 | 0.0705 | - | - | 0.0907 | - | - |
|  | 753 | 0.0042* | - | - | 0.0814 | - | - |

*significant
